# Wear Behavior and Surface Quality of Dental Bioactive Ions-Releasing Resins Under Simulated Chewing Conditions

**DOI:** 10.3389/froh.2021.628026

**Published:** 2021-02-12

**Authors:** Isadora Martini Garcia, Abdulrahman A. Balhaddad, Noorhan Aljuboori, Maria Salem Ibrahim, Lamia Mokeem, Akudo Ogubunka, Fabrício Mezzomo Collares, Mary Anne Sampaio de Melo

**Affiliations:** ^1^Department of Dental Materials, School of Dentistry, Federal University of Rio Grande do Sul, Porto Alegre, Brazil; ^2^Ph.D. Program in Dental Biomedical Sciences, University of Maryland School of Dentistry, Baltimore, MD, United States; ^3^Department of Restorative Dental Sciences, College of Dentistry, Imam Abdulrahman Bin Faisal University, Dammam, Saudi Arabia; ^4^Division of Operative Dentistry, Department of General Dentistry, University of Maryland School of Dentistry, Baltimore, MD, United States; ^5^Department of Preventive Dental Sciences, College of Dentistry, Imam Abdulrahman Bin Faisal University, Dammam, Saudi Arabia

**Keywords:** polymers, dental caries, composite resins, fluorides wear behavior of dental bioactive resins, ion releasing

## Abstract

Bioactive materials can reduce caries lesions on the marginal sealed teeth by providing the release of ions, such as calcium, phosphate, fluoride, zinc, magnesium, and strontium. The presence of such ions affects the dissolution balance of hydroxyapatite, nucleation, and epitaxial growth of its crystals. Previous studies mostly focused on the ion-releasing behavior of bioactive materials. Little is known about their wear behavior sealed tooth under mastication. This study aimed to evaluate the wear behavior and surface quality of dental bioactive resins under a simulated chewing model and compare them with a resin without bioactive agents. Three bioactive resins (Activa, BioCoat, and Beautifil Flow-Plus) were investigated. A resin composite without bioactive agents was used as a control group. Each resin was applied to the occlusal surface of extracted molars and subjected to *in vitro* chewing simulation model. We have assessed the average surface roughness (Ra), maximum high of the profile (Rt), and maximum valley depth (Rv) before and after the chewing simulation model. Vickers hardness and scanning electron microscopy (SEM) also analyzed the final material surface quality). Overall, all groups had increased surface roughness after chewing simulation. SEM analysis revealed a similar pattern among the materials. However, the resin with polymeric microcapsules doped with bioactive agents (BioCoat) showed increased surface roughness parameters. The material with Surface Pre-reacted Glass Ionomer (Beautifil Flow-Plus) showed no differences compared to the control group and improved microhardness. The addition of bioactive agents may influence surface properties, impairing resin composites' functional and biological properties. Future studies are encouraged to analyze bioactive resin composites under high chemical and biological challenges *in vitro* with pH cycles or *in situ* models.

## Introduction

Dental caries is the most prevalent chronic disease worldwide, affecting 60–90% of children [1]. Oral health preventive guidelines widely recommend sealing the tooth surface as a non-invasive preventive approach [2]. Dental sealants' application over the tooth surface is painless, fast, and welcomed by children [3]. Caries lesions on the marginal sealed teeth can be initiated by bacterial acids resulting in the dissolution of hydroxyapatite crystals with calcium (Ca^2+^) and phosphate (${\text{PO}}_{4}^{3-}$) ions loss [4, 5].

Bioactive resin sealants could be one of the most desirable approaches for managing caries due to the potential of providing localized ion release near the tooth surface [6]. The bioactivity of these materials can be attributed to the ionic exchange with saliva and tooth structure. The material's interactivity with the surrounding microenvironment would help maintain healthy teeth [6–8]. Bioactive resins are expected to provide essential ions, such as calcium and phosphate, to restore the physiological equilibrium between tooth minerals and oral fluids [9, 10]. These materials claim continuously recharge the ionic components of saliva, teeth, and the material itself. New dental materials have been developed to present biointeractivity with dental tissues to prevent caries' recurrence around sealed or restored teeth [6, 9–12].

In the oral environment, dental materials face many challenges to their long-term service. Fatigue wear due to the chewing process's cyclic nature causes degradation of dental materials [13]. Dental resins show a particular wear pattern because their composition's characteristics directly affect their wear resistance [14, 15]. Bioactive resins are relatively new in the dental market. Many manufacturers have intentionally added different ion releasing-sources in a range of size and concentration as an anticaries component [16–18]. The variation in composition, solubility, and permeability of the bioactive resin is intended to maximize and sustain the ion release over time [10, 19, 20]. However, changes in the resin formulations to facilitate ion releasing may impact wear behavior making the material prone to physical modifications under masticatory load [18]. Few studies investigated bioactive fillers' effects in dental resins under high-challenge situations [18, 21, 22]. In this context, it is essential to consider that the oral environment and masticatory loads can induce high surface roughness, contributing to biofilm formation on the resin surface [23]. Over time, the damage on material surface ‘s properties can jeopardize the plaque removal and induce a higher biofilm development on the material.”

In this study, bioactive resins with low viscosity were applied to human teeth' occlusal surfaces to be subjected to chewing simulation and surface morphology analysis. This study aimed to evaluate the wear behavior and surface quality of dental bioactive resins under a simulated chewing model and compare them with a resin without bioactive agents.

## Materials and Methods

### Study Design

**Figure 1:**
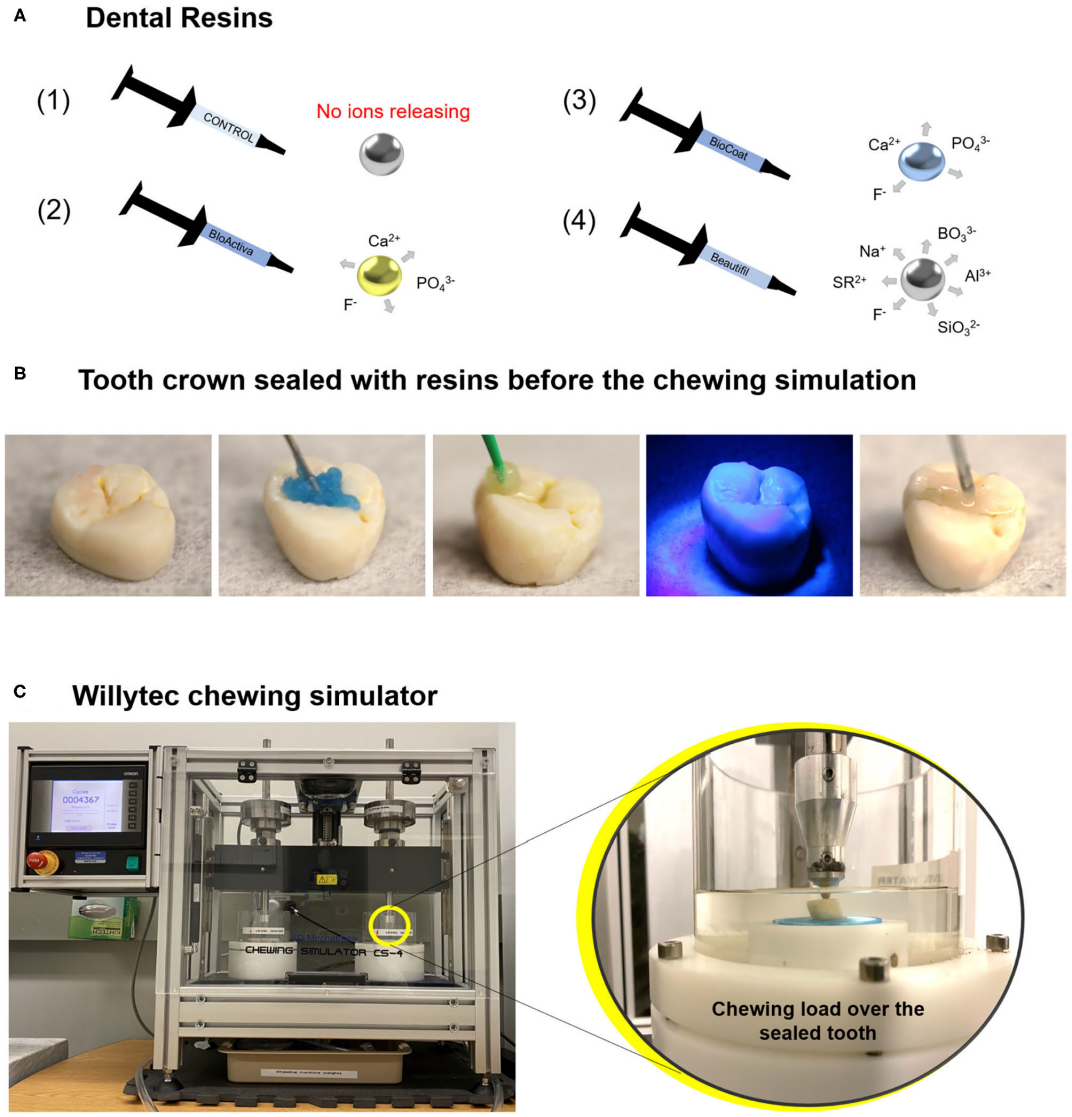
Image **(A)** displays an illustration of the dental resins applied in this study: bioactive resins, with an ion-releasing composition (schematic draw from 2 to 4), and a control group without ion-releasing composition (schematic draw 1). A chewing simulation machine used in this study and a representative sealed tooth with a bioactive resin on the chewing surface. Image **(B)** shows a tooth crown sealed with a dental resin before the chewing simulation. Image **(C)** displays the chewing simulation machine. The magnification shows the tooth crown sealed with the dental resin positioned in the chewing simulation machine.

This study employed a completely randomized, single-blind experimental design with 10 experimental units (sealed tooth) per group. Using a computer-generated list, we randomly assigned the teeth to one of four groups, as showed in Figure 1A.

The groups are described as follows: (1) A dental resin without bioactive materials was included as a control group (TPH 3 Flow, Dentsply Sirona, Milford, DE, USA), (2) Activa BioRestorative (Pulpdent, Watertown, MA, USA), (3) BioCoat (with SmartCap Technology, Plymouth Meeting, PA, USA), and (4) Beautifil Flow Plus [with Surface Pre-reacted Glass Ionomer (S-PRG) SHOFU Inc., Kyoto, Japan]. A detailed description of the composition of tested materials is displayed in Table 1. The response variables were average surface roughness (Ra), maximum high of the profile (Rt), and maximum valley depth (Rv) before and after the chewing simulation model (expressed in micrometers), surface hardness values, and surface morphology via scanning electron microscopy (SEM).

**Table 1:** Composition and manufacturer of the three bioactive resin composites and the control group without bioactive agents provided by the Safety Data Sheet of each manufacturer.

**Identification**	**Resin composite**	**Manufacturer**	**Composition (%)**
Bioactive	Activa BioActive-Restorative (Bioactive ionic resin with reactive glass filler)	Pulpdent, Watertown, MA, USA	Blend of diurethane and other methacrylates with modified polyacrylic acid (44.6%), Silica, amorphous (6.7%), Sodium fluoride (0.75%).
BioCoat	BioCoat Bioactive Pit and Fissure Sealant	Premier Dental Products Company, Plymouth Meeting, PA, USA	Triethylene glycol dimethacrylates, (1-methylethylidene)bis[4,1-phenyleneoxy(2-hydroxy-3,1-propanediyl)]bismethacrylate, Fumed Silica, Barrium Aluminoborosilicate (≤ 60%); Calcium Donor (≤ 2%); Photo-Initiator ≤ 2.5.
Beautifil	Beautifil Flow Plus	Shofu Inc., San Marcos CA, USA	Bisphenol A*-*glycidyl methacrylate (~15–25%), Triethylenglycol dimethacrylates (~12–14%), Aluminofluoro-borosilicate glass (~50–60%), Al_2_O_3_ (1–2%), Camphorquinone.
Control	TPH 3 Flow	Dentsply Caulk, Milford, DE, USA	Bisphenol A-glycidyl methacrylate (<20%), Urethane modified Bis-GMA dimethacrylates (<10%), Polymerizable dimethacrylate resins (<25%), Silicon Dioxide—Amorphous (<10%), Barium boron fluoro alumino silicate glass (<70%).

### Sample Preparation

The Institutional Research Board at the University of Maryland HP-00079029/approved the use of extracted human teeth for this investigation. The teeth were refrigerated at 4°C in a 0.01% (w/v) thymol solution until use. Next, using a water-cooled diamond saw and a cutting machine (IsoMet Low-Speed Saw, Buehler, Lake Bluff, IL), the teeth were cut 2 mm below the cement–enamel junction to remove the roots. The tooth crowns were inspected under ×10 magnification to ensure an occlusal surface free of cracks or defects (Leica Zoom 2000—Leica Microsystems GmbH, Wetzlar, Germany).

Phosphoric acid at 37°C was applied on the enamel of occlusal areas for 15 s, rinsed with distilled water for 15 s, and spray dried. The bonding agent (OptiBond Solo Plus, Kerr, Brea, CA) was applied using a micro brush and light-cured for 20 s. Each resin was applied on the occlusal surfaces of the teeth, a Mylar strip was placed on the top of the etched enamel, and the material was photoactivated for 20 s with a light-emitting diode with 1,200 mW/cm^2^ (Valo grand, Ultradent Products Inc., South Jordan, UT, USA). The occlusal area was covered entirely to obtain flat surfaces. Figure 1B displays the application of the bioactive resin over the tooth surface.

### Chewing Simulation Model

Extracted teeth sealed with the tested bioactive and control resins were subjected to simulated chewing using a chewing simulator (Chewing simulator CS-4, SD Mechatronik GMBH, Feldkirchen-Westerham, Bavaria, Germany). The machine used in this study described in Figure 1C is a dual-axis chewing simulator that allows appropriate standardization of the number of cycles, load, speed, and frequency [24]. Artificial saliva was used as a lubricant medium during the chewing simulation [25]. The lubricant acts to simulate a moist oral environment and help remove wear debris generated during the chewing. The saliva solution was prepared with 1 L of distilled water and 0.1029 g of CaCl_2_.2H_2_O, 0.04066 g of MgCl_2_, 0.544 g of KH_2_PO_4_, 4.766 g of Hepes, 2.2365 g of KCl. The solution was mixed, and the pH was adjusted to 7 [26].

The sealed teeth were subject to a unidirectional load of 49 N, an equivalent of 5 kg, and 80,000 cycles, equivalent to almost 4 months of clinical service [24]. The upward movement was 2 mm, downward movement of 1 mm, horizontal movement of 0.7 mm, speed of upward movement of 40 mm/s, speed of downward movement of 40 mm/s, speed of horizontal movement of 40 mm/s, frequency of 1 Hz. The antagonist was a steatite tip with 6 mm of diameter, and the direction was forwards under load, backward without load.

### Assessment of the Tooth Wear

Each resin's surface wear behavior before and after the chewing simulation was analyzed using a contact profilometer (Surftest SJ-310, Mitutoyo America, Aurora, IL, USA). For this purpose, five measurements were performed at the center of each occlusal area. Standard roughness calibration specimen was used for calibration. The measurements were performed with a stylus tip of 5 μm at 0.5 mm/s. A force of 4 mN was applied with a cut-off of 0.25 and 1.5-mm tracing length [27]. The following parameters were assessed:

#### Average Surface Roughness (Ra)

Ra represents the arithmetical mean of the profile deviations' absolute values from the roughness profile's mean line.

#### Maximum High of the Profile (Rt)

Rt measures the difference between the maximum peak height and the maximum valley depth. Rt is used to detect any measure discrepancy in the surface of the sample.

#### Maximum Valley Depth (Rv)

Rv measures the deepest valley produced by the chewing simulation. A high Rv value means a high amount of wear.

With the measurements pre- and post-chewing simulation, it was possible to analyze Ra, Rv, and Rt's variation for each tooth and each group (ΔRa, ΔRv, and ΔRt). The variation (delta-Δ) was calculated via subtracting the final values minus the initial ones. The results were expressed in μm.

### Vickers Hardness (Indentation Modulus) of Bioactive Resins After Chewing

Besides wear and surface roughness evaluations, the resins were assessed via indentation modulus after chewing simulation. The Vickers hardness test method allows for a rapid and precise assessment of the material's resistance to deformation. This method consists of indenting the test material with a diamond indenter, a pyramid with a square base, and an angle of 136° between opposite faces subjected to a test force of between 1 gf and 100 kgf [28]. Equation (1) was used to calculate the Vickers hardness:


(1) $$\begin{array}{r} HV=\frac{F}{A}=\;\frac{2F.\sin\frac{136^{{}^{\circ}}}{2}}{d^{2}} \end{array}$$

In which “F” is the applied force, and “A” is the printed square area on the polymer surface calculated by measuring its diagonals.

Five samples of each per group were analyzed, and the measurements were performed using a Vickers indenter with 25 g for 10 s (microhardness tester, HMV-G, Shimadzu Corp., Tokyo, Japan). Five indentations were completed on each resin on the teeth. The average hardness value for each resin per group was calculated.

### Analysis of Surface Morphology *via* Scanning Electron Microscopy (SEM)

One sample per group was qualitatively analyzed via SEM. Each representative sample was mounted on aluminum stubs with carbon conductive double-face adhesive tapes and sputter-coated with 10–20 nm of platinum/Palladium in a sputter coater (EMS 150T ES, Electron Microscopy Sciences, PA). SEM images were taken in a scanning electron microscope (Quanta 200 FEI Company, Hillsboro, OR, USA) under 100 and 2,000× magnification.

### Statistical Analysis

The software SigmaPlot, version 12.0 (Systat Software, Inc., San Jose, CA, USA), was used to analyze the data. Initially, the assumptions of equality of variances (Levene's test) and the data distribution (the Shapiro-Wilk test) were verified.

The difference between pre- and post-Ra, Rt, or Rv within each group was analyzed via paired *t*-tests. For comparison among the groups within pre-reading and post-reading data, pre-Ra and pre-Rt were compared via Kruskal-Wallis, while pre-Rv was compared via One-Way ANOVA. When a significant difference was noted, Dunn's test was used as *post-hoc* for Kruskal-Wallis, while Tukey's test was applied as *post-hoc* for One-Way ANOVA.

Post-Ra, post-Rt, and post-Rv were also analyzed among the groups of resins. Post-Ra was analyzed via One-Way ANOVA with Tukey's *post-hoc* test. Post-Rt and post-Rv were analyzed via Kruskal-Wallis, and Dunn's test was applied when there was a significant difference among groups.

ΔRa was analyzed among groups via One-Way ANOVA and Tukey's test, ΔRt was analyzed among groups via One-Way ANOVA, and ΔRv was analyzed among groups via Kruskal-Wallis. The Vickers hardness was tested via One-Way ANOVA and Tukey's test among groups. A significance level of 0.05 was considered for all tests.

## Results

**Figure 2:**
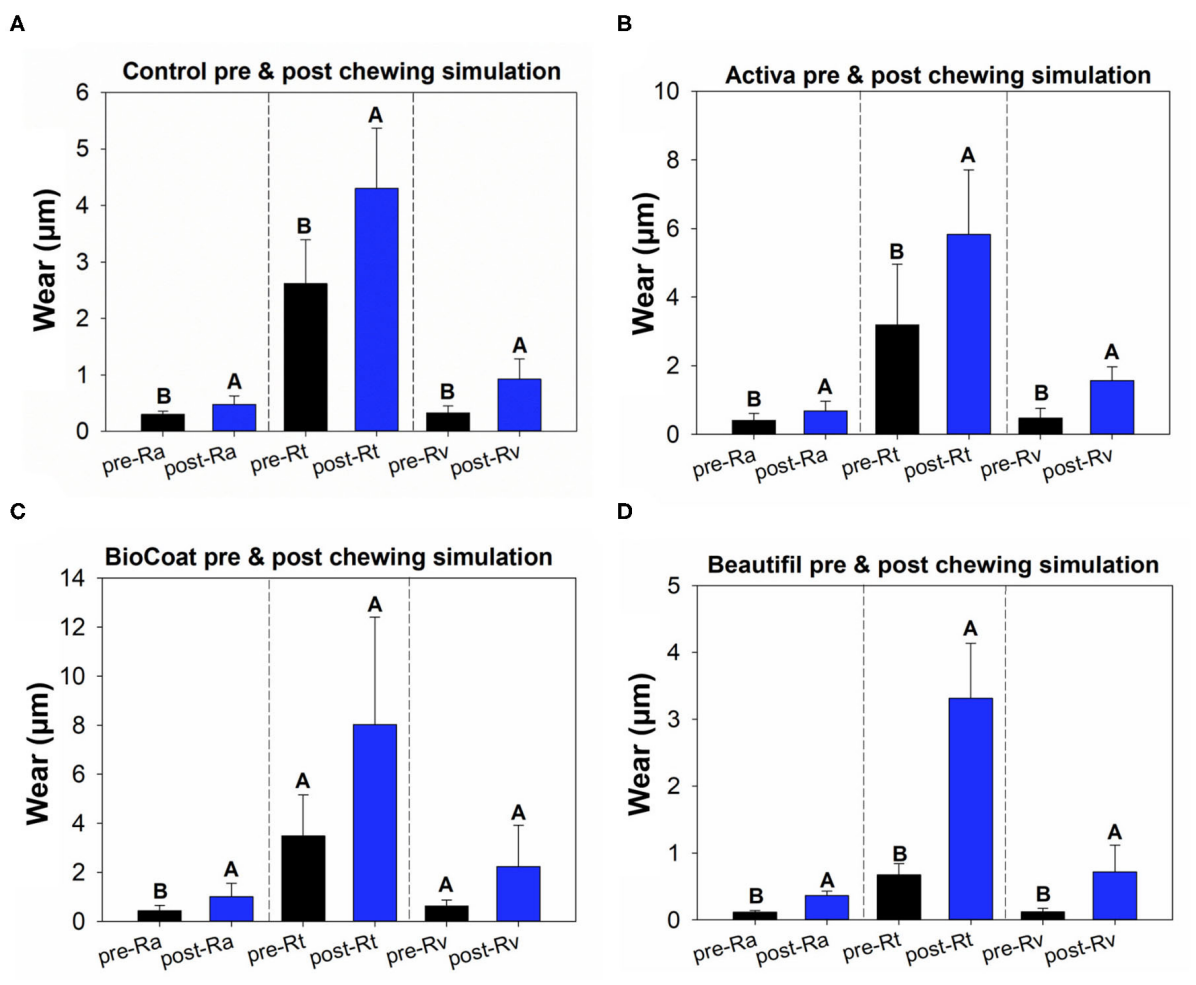
Results of tooth wear assessment. The data of pre- and post-surface via paired *t*-tests. Image **(A)** displays the results of pre- and post-Ra, Rt, and Rv for the control group, the TPH bioactive resin. Image **(B)** displays the results of pre- and post-Ra, Rt, and Rv for the Activa bioactive resin. Image **(C)** displays the results of pre- and post-Ra, Rt, and Rv for the BioCoat bioactive resin. Image **(D)** displays the results of pre- and post-Ra, Rt, and Rv for the Beautifil Flow-Plus bioactive resin. Different letters indicate a statistically significant difference between pre- and post-Ra, or pre- and post-Rt, or pre- and post-Rv within each group (*p* < 0.05).

In the roughness analysis, all groups of bioactive resins showed higher Ra and Rv after the chewing simulation compared to the initial values (*p* < 0.05) (Figure 2). The roughness parameter Rt also showed significantly higher values after the chewing simulation for the control group, Activa, and Beautifil groups (*p* < 0.05). The resin BioCoat showed no statistically significant difference between pre and post-Rt (*p* > 0.05).

**Figure 3:**
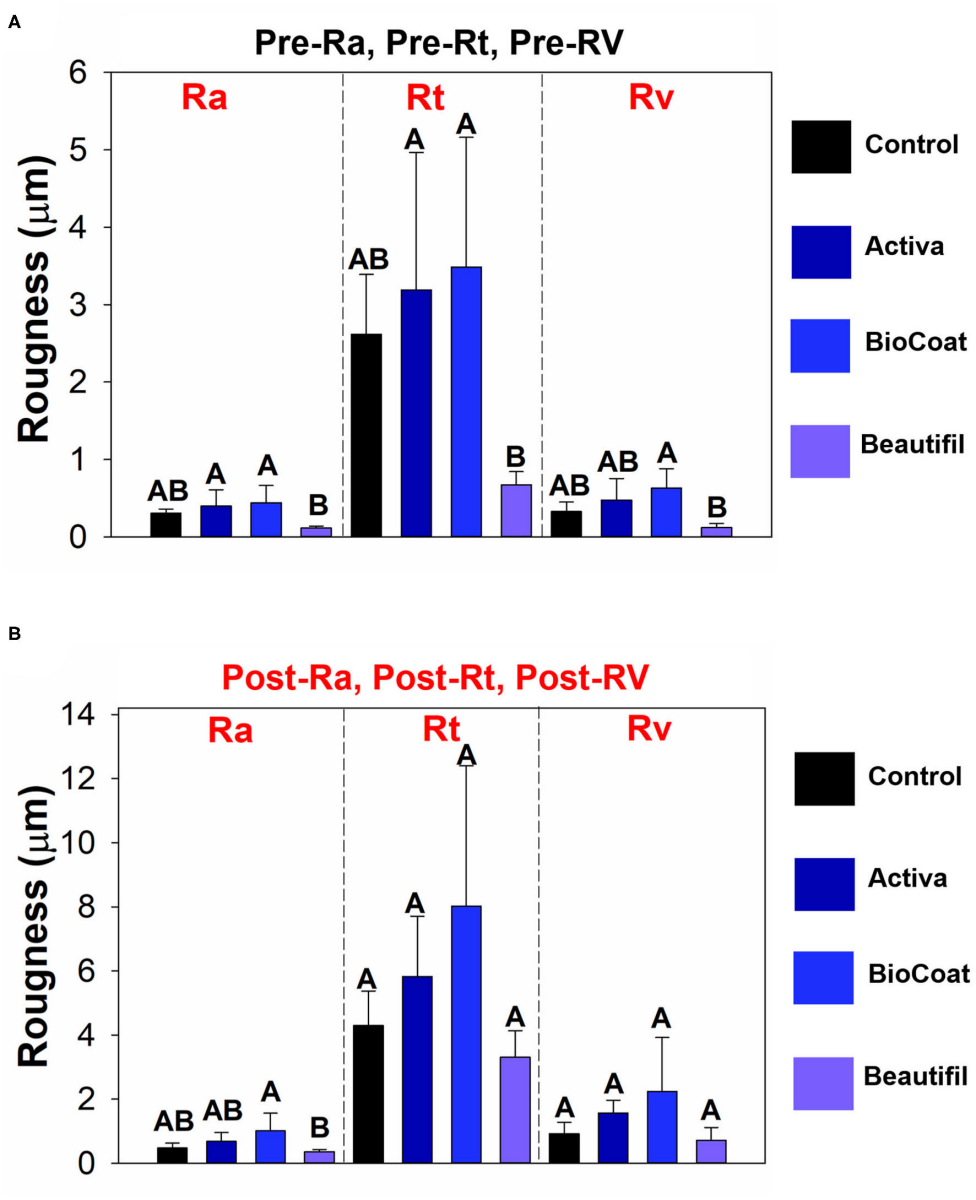
Results of tooth wear assessment. The data of pre- or post-surface roughness parameters among groups of bioactive resins were compared via One-Way ANOVA or Kruskall-Wallis. *Post*-*hoc* tests were applied when a significant difference was noted. Image **(A)** displays the results of pre-Ra, pre-Rt, and pre-Rv among groups. Different letters indicate statistically significant differences among groups of bioactive resins within each surface roughness parameter (pre-Ra, or pre-Rt, or pre-Rv) (*p* < 0.05). Image **(B)** displays the results of post-Ra, post-Rt, and post-Rv among groups. Different letters indicate statistically significant differences among groups of bioactive resins within each surface roughness parameter (post-Ra, or post-Rt, or post-Rv) (*p* < 0.05).

The resin composite Beautifil showed lower surface roughness in comparison to BioCoat for all three surface parameters (*p* < 0.05), and there was no difference among the control, Activa, and BioCoat groups in Pre-Ra, pre-Rt, and pre-Rv (*p* > 0.05) (Figure 3). Post-Ra, post-Rt, and post-Rv showed different behavior, with a statistical difference among groups only for post-Ra (*p* < 0.05).

**Figure 4:**
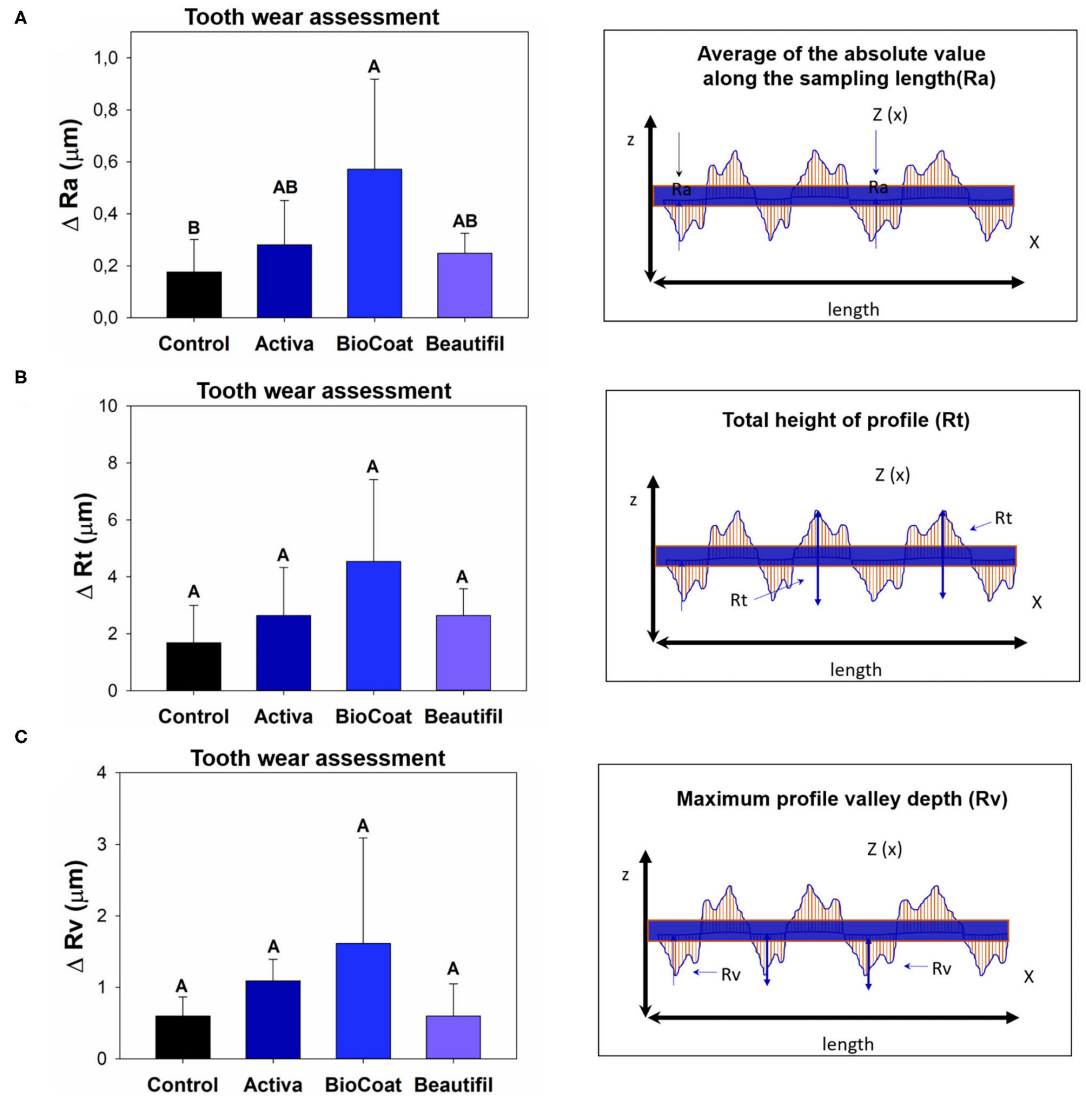
Results of tooth wear assessment via surface roughness variation (Δ = delta calculation). The data for each roughness parameter (Ra, Rt, or Rv) were compared via One-Way ANOVA or Kruskall-Wallis. *Post*-*hoc* tests were applied when a significant difference was noted. Image **(A)** shows the difference of ΔRa among groups. Image **(B)** shows the difference of ΔRt among groups. Image **(C)** shows the difference of ΔRv among groups. Different letters indicate statistically significant differences among groups within each surface roughness parameter (Δ-Ra, or Δ-Rt, or Δ-Rv) (*p* < 0.05).

The difference between pre-and post-surface roughness parameters is expressed as ΔRa, ΔRt, and ΔRv in Figure 4. There was a statistically significant difference among groups for ΔRa (*p* < 0.05). In this analysis, the control group TPH, which was the only bioactive resin without bioactive fillers, showed no statistical difference for Activa and Beautifil (*p* > 0.05). The group BioCoat had higher ΔRa than the control group (*p* < 0.05). There were no differences among groups for ΔRt or ΔRv (*p* > 0.05). However, it was clear that the BioCoat group showed the highest standard-deviation values among the resins.

**Figure 5:**
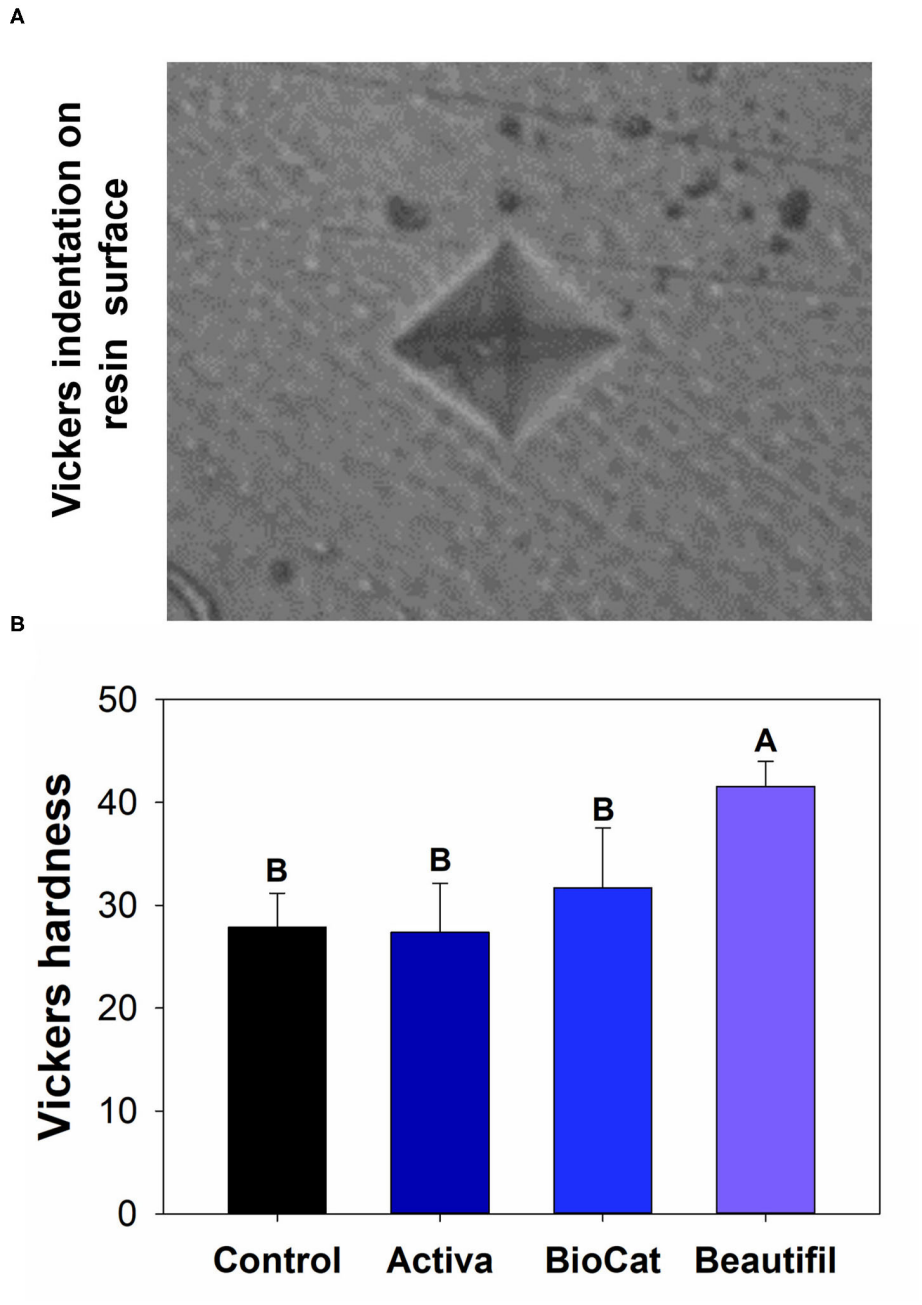
**(A)** Optical image of a standard Vickers indentation on the composite surface. **(B)** Means and standard values for the Vickers surface hardness for all tested groups. Different letters indicate statistically significant difference among groups for Vickers hardness (*p* < 0.05).

There was a statistically significant difference among groups considering the Vickers hardness (*p* < 0.05) (Figure 5). The values ranged from 27.36 (±4.77) for Activa to 41.56 (±2.44) for Beautifil (*p* < 0.05). The control group, Activa, and BioCoat showed no differences for Vickers hardness (*p* > 0.05), and all these three bioactive resins presented lower results than Beautifil (*p* < 0.05).

**Figure 6:**
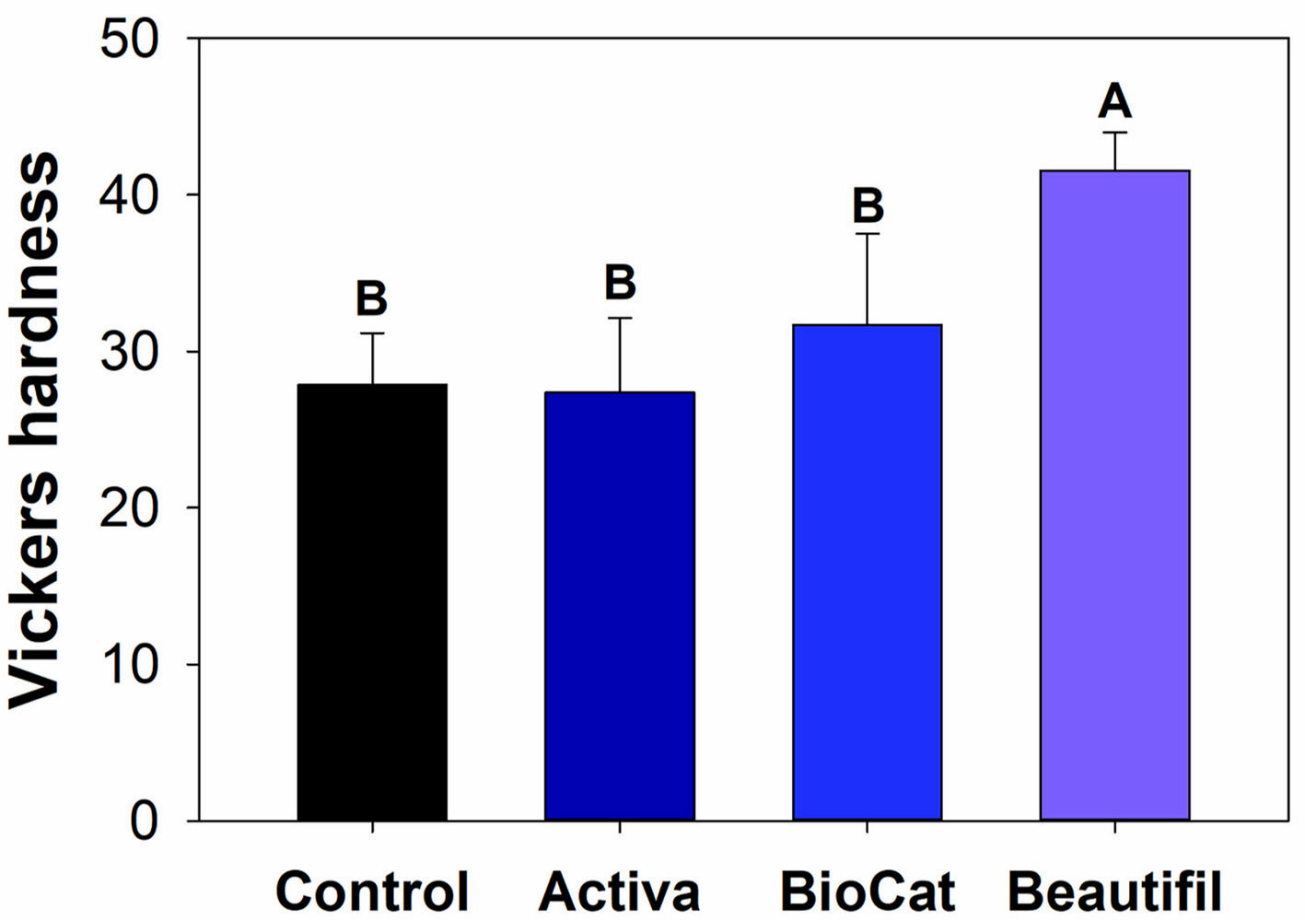
Representative scanning electron microscopy (SEM) images of the control group and bioactive resins: Activa, BioCoat, and Beautifil. Upper images were acquired with 100× magnification. Images on the bottom were acquired with 2,000× magnification.

Regarding the surface morphology (Figure 6), all groups exhibited a characteristic surface alteration in the contact area during the chewing simulation, as observed in the images at 100× magnification. More defects with larger cracks are observed for the control and Activa groups, following by Beautifil and Biocoat. However, all groups presented an irregular morphology identified with the highest magnification (2,000×).

## Discussion

The main advantage of bioactive resins is to drive an ecological shift inside biofilms toward non-dysbiotic conditions. Therefore, a higher pH and ions' availability could reduce the chances of bacteria adherence, biofilm formation and increase dental remineralization chances [29]. However, the wear phenomenon may change resins' surface properties over time. The cyclic wear phenomenon of dental materials is a gradual material removal from the restored surface due to the interaction of teeth against each other during movement [15]. Clinical wear is a complex process involving two-and-three-body wear and is influenced by materials' composition, antagonist properties, diet, and applied force [15]. Dental materials exhibit different wear mechanisms while underwear conditions *in vitro* [30, 31]. None of these standing mechanisms can completely simulate the clinical wear process [30]. Consequently, a straight correlation between the clinical and *in vitro* wear tests remains a challenge [32].

Therefore, *in vitro* studies on wear aim to rank restorative materials about their wear resistance and understand the wear behavior under particular simulated clinical conditions [33]. Simulating clinical settings, such as masticatory load and saliva, the *in vitro* chewing testings provide valuable inputs on planning new products and evaluating materials. In the current research, a Willytec simulator was set up to exert particular wear mimicking ~4 months of occlusal movements over the sealed teeth.

As an outcome, the Beautifil group showed improved outcomes compared to Activa and Bioactive, with lower roughness before or after the chewing simulation, and without differences for the control group (Figure 3). Here, the tested materials differ regarding the composition and their content (Table 1). For instance, resin-based material comprises a three-phase system, mainly composed of filler particles, resin matrix, and the coupling agent used to bond the first two elements [34]. According to the manufacturer, Beautifil is considered a Giomer because it has a high content of surface pre-reacted glass ionomer (S-PRG) particles with 0.01–4 μm, able to release and recharge fluoride. Although all groups contain dimethacrylates and inorganic filler in their composition, the wear resistance performance of Beautifil may be due to an enhanced filler distribution on the resin matrix or a difference in the filler size leading to homogeneity and maintenance of surface properties.

The parameter Rv, which measures the deepest valley, was increased for all groups after chewing simulation (Figure 2), suggesting a loss of material on the surface for the control, Activa, BioCoat, and Beautifil. Even though there was no difference for ΔRv regardless of the bioactive resin group (Figure 4), BioCoat, showed a high standard deviation value (mean ± SD = 1.61 ± 1.48 μm). The high variation for this group may be related to its wear process, and consequently, loss of particles or resin itself. The SEM images of the worn surfaces are suggestive of wear scars and debris.

Similarly, the Rt parameter indicates the difference between the maximum peak height and the maximum valley depth, increased for the control, Activa, and Beautifil. At the same time, BioCoat did not show a statistical difference between pre and post-chewing. The high standard-deviation value for this bioactive resin in Rv and Rt probably reflects the discrepancies in BioCoat surface roughness (Figure 2). Like the Rv outcome, ΔRt also showed a high standard-deviation (Figure 4), suggesting that the material probably offers an irregular surface.

The evaluation of roughness average (Ra) is a common approach to test the surface of dental materials and how the environment impacts their surface's properties [35]. An increased roughness is usually more prone to microorganisms colonization and biofilm formation [18, 36, 37]. Bioactive resins, such as those tested here, will contact the enamel inside the mouth. The increased roughness may be a challenge for hygiene control, negatively impacting caries prevention. Therefore, the maintenance of roughness is also crucial for biofilm control. In this study, all groups showed a high initial roughness. The Mylar strip before resins' photoactivation during sample preparation should have helped achieve the smoothest possible surface. Different bioactive resins previously showed varied roughness depending, for instance, on their quantity of fillers [22, 38]. The fillers' heterogeneity, content, size, or shape probably led to the present study's high roughness. It is noteworthy that Beautifil resin presented lower initial and final Ra compared to Activa and BioCoat. This resin had no difference for the control group (resin without bioactive fillers). As aforementioned, the fillers' properties and the composites' solubility may differ among groups, leading to higher wear and Ra for Activa and BioCoat.

Beautifil contains S-PRG particles, which are bioactive fillers able to release ions, mainly boron and fluoride [39, 40]. Activa releases and recharges calcium, phosphate, and fluoride, while BioCoat has resin-based microcapsules filled with ionic solutions of fluoride, calcium, and phosphate [41]. Beautifil may show better smoothness compared to BioCoat and Activa but also has superior physicochemical stability. Therefore, the initial roughness is decreased for this resin, and the variation and the final roughness. On the other hand, the polymeric microcapsules of BioCoat may are not well-distributed as inorganic fillers of the other bioactive resins and may have released the bioactive agents from the core during the chewing simulation, increasing the variation intragroup. The variation that occurs depending on the resins' chemical formulation may have contributed to the non-normal distribution of data for some parameters.

Interestingly, a previous study [18] investigated another resin from the same brand, Beautifil II, containing S-PRG particles. The authors observed a higher porosity and roughness for Beautifil II than a conventional glass-ionomer cement (Fuji IX GP EXTRA, Tokyo, Japan) and a conventional resin (Herculite XRV Ultra, Kerr, Orange, CA, USA). The higher porosity of Beautifil was even pronounced when the materials were immersed in lactic acid, a standard caries-related bacterial end product of sugar metabolism.

After the chewing simulation, the resins were also analyzed via microhardness and SEM. There was no statistical difference among the control group, Activa, and BioCoat, with significantly higher values for Beautifil (Figure 5). The resin-based materials' hardness depends on materials chemical properties, such as the degree of conversion of carbon-carbon double bonds of methacrylate groups and crosslinking density. Moreover, the filler content and distribution on the surface influences materials' hardness. It is not possible to assure if the difference found was due to S-PRG incorporation, total inorganic content, or variation of polymeric chains. The Beautifil group showed a roughness compatible with the control group before and after the chewing simulation and a superior microhardness compared to the other bioactive resins.

SEM analysis was performed in one sample per group as qualitative and additional information. Interestingly, in the analyzed samples, BioCoat did not present defects, such as the other groups. This result can be related to the higher standard-deviation values for this group than the control and the other bioactive resins. A deeper understanding of these bioactive resins under cariogenic challenges could help plan and develop therapeutic materials.

This study selected three bioactive materials to evaluate, although an increasing number of bioactive materials exist in the market. Therefore, readers must be aware of over-generalizations since we understand that resin composites formulations are highly heterogeneous. Also, we applied 80,000 cycles during the loading. However, the cycle range used in the literature can vary. Different loading cycles within the wide range of 5,000–1,200,000 cycles are described in the literature as ideal for the initial simulation of *in vivo* chewing [42]. Moreover, even though the artificial saliva used has been reported in a previous study [26], sterilized saliva or mucin-containing saliva could be applied to analyze the wear under loading. More complex saliva could better mimic the oral environment, mainly because of the ions exchange and wear process complexity under chewing simulation.

## Conclusion

In this *in vitro* study, the use of the two-body wear simulation model revealed initial information on ion-releasing bioactive resins' wear behavior for the first time. The bioactive resin containing S-PRG particles did not differ from the control and showed the highest microhardness. Overall, bioactive resins may not have inferior wear behavior and surface quality compared to non-bioactive conventional resin. Future studies investigating the filler's content in the filler particles' matrix and size incorporated into bioactive resins are encouraged.

## Data Availability Statement

The original contributions generated for the study are included in the article/supplementary material, further inquiries can be directed to the corresponding author/s.

## Ethics Statement

The studies involving human participants were reviewed and approved by University of Maryland Baltimore Institutional Research Board. Written informed consent for participation was not required for this study in accordance with the national legislation and the institutional requirements.

## Author Contributions

IG and AB processed the experimental data, performed the analysis, drafted the manuscript, and designed the figures. NA, MI, LM, and AO performed the measurements. FC and MdM were involved in planning, supervising the work, and interpreting the results and worked on the manuscript. All authors contributed to the article and approved the submitted version.

## Conflict of Interest

The authors declare that the research was conducted in the absence of any commercial or financial relationships that could be construed as a potential conflict of interest.
